# Impact of Asthma on the Quality of Sleep in Young People

**DOI:** 10.7759/cureus.16098

**Published:** 2021-07-01

**Authors:** Akhtar Ali, Deepa Kumari, Deepak Kataria, FNU Priyanka, Muhammad Umair Nawaz, FNU Pariya, Rama Kalyani Kavuri, Sidra Naz, Amna Jamil, Faizan Shaukat

**Affiliations:** 1 Pulmonology and Critical Care, Services Hospital, Lahore, PAK; 2 Internal Medicine, Chandka Medical College, Larkana, PAK; 3 Internal Medicine, Shaheed Mohtarma Benazir Bhutto Medical University, Larkana, PAK; 4 Internal Medicine, Jinnah Sindh Medical University, Karachi, PAK; 5 Internal Medicine, Dr. Vasantrao Pawar Medical College Hospital & Research Centre, Nashik, IND; 6 Internal Medicine, University of Health Sciences, Lahore, PAK; 7 Internal Medicine, Jinnah Postgraduate Medical Centre, Karachi, PAK; 8 Internal Medicine, Dow University of Health Sciences, Karachi, PAK

**Keywords:** sleep quality, asthma, young patients, quality of life, impact

## Abstract

Introduction

Asthma has a negative impact on the quality of life of patients and their families. One of the factors responsible for the low quality of life is poor sleep quality in asthmatic patients. Sleep disturbances, such as difficulty initiating and maintaining sleep, are common in asthma. In this study, we aim to determine the quality of sleep in young asthmatic patients in a local setting.

Method

This case-control study was conducted in the pulmonology and internal medicine unit of a tertiary care hospital, Pakistan from January 2021 to May 2021. After seeking informed consent, 200 patients with a previously confirmed diagnosis of asthma were enrolled in the study. The control group also included 200 participants. Pittsburgh Sleep Quality Index (PSQI) is an efficient measure of the quality and pattern of sleep. A global PSQI score of ≥5 signifies “poor sleep quality.”

Results

The mean PSQI score was significantly higher in the asthmatic group compared to the control group (6.26 ± 2.01 vs. 3.41 ± 0.50; p-value: <0.0001). The percentage of participants with a PSQI score of ≥5 was significantly higher in the asthmatic group compared to the control group (54.5% vs. 17.0%; p-value: <0.0001).

Conclusion

Sleep disturbance is very common in young patients with asthma. Poor sleep may interfere with their daily performance, which may further have a negative impact on the quality of life in asthmatic patients. Management of asthma should also include improving sleep quality.

## Introduction

Asthma is a chronic obstructive disease of the respiratory tract [1]. The global burden of asthma has increased over the last two decades whereby it now affects approximately 300 million people worldwide, with a predilection towards industrialized countries. The onset of the disease is mostly early in life, with almost 90% of cases diagnosed before the age of six years [1,2]. It is characterized by the presence of hyper-responsive airways with reversible airway obstruction and inflammation, leading to symptoms of cough, wheeze, breathlessness, and chest tightness [3,4].

Asthma has a great impact on the quality of life (QOL) of patients and their families. The magnitude of this morbidity is affected by several personal factors [5]. One of these factors responsible for the low QOL is poor sleep quality in asthmatic patients. Sleep disturbance includes difficulty initiating and maintaining sleep; patients with asthma commonly report frequent awakenings [6-9]. Although nocturnal exacerbations can indicate inadequate asthma control and disturbed sleep, poor sleep has been reported in patients with well-controlled asthma, suggesting that poor sleep may be independent of nocturnal asthma symptoms [6,10].

There are very limited data available in the local and regional settings to assess the impact of asthma on the quality of sleep. Therefore, we aim to determine the impact of asthma on the quality of sleep in asthmatic patients.

## Materials and methods

This case-control study was conducted from January 2021 to May 2021. Participants were enrolled from the pulmonology and internal medicine unit of a tertiary care hospital, Pakistan. Informed consent was taken from the participants. Patients with a previously confirmed diagnosis of asthma, of both gender and aged between 10 and 24 years, were enrolled as the study group (n=200). Another 200 participants were enrolled in the study as the control group from the outpatient department. Ethical approval was taken from the institutional review board before enrolling the participants.

Pittsburgh Sleep Quality Index (PSQI) is an efficient measure of the quality and pattern of sleep. It assesses sleep quality on seven components, i.e. subjective sleep quality, sleep latency, sleep duration, habitual sleep efficiency, sleep disturbances, the use of sleeping medication, and daytime dysfunction. Its scores range from a minimum zero to a maximum of 21. The combined score of all seven components is termed as “global score of PSQI” and a global PSQI score of ≥5 signifies “poor sleep quality.” Its internal consistency and reliability coefficient (Cronbach's alpha) is 0.83 for its seven components [11]. The PSQI score of participants of both groups was compared.

Data were entered and analyzed using the Statistical Package for the Social Sciences for Windows, version 22.0 (SPSS, IBM Corp., Armonk, NY, USA). Patients’ demographics were calculated as frequencies and percentages. Numerical data were presented as mean and standard deviation. The comparison of the mean PSQI score between the two groups was done by applying a t-test. Similarly, the comparison of participants with PSQI square equal to more than 5 was done by applying chi-square. A p-value of less than 0.05 was considered significant.

## Results

The mean age of participants in the case group was 14 ± 4 years, while in the control group it was 15 ± 4 years. The characteristics, such as age and gender, were comparable between both groups (Table 1).

**Table 1:** The demographics of the participants NS: Not Significant

Characteristics	Case group (n=200)	Control group (n=200)	P-value
Age groups (in years)			
10-17	109 (54.5%)	107 (53.5%)	NS
18-24	91 (45.5%)	93 (46.5%)	
Gender			
Male	111 (55.5%)	109 (54.5%)	NS
Female	89 (44.5%)	91 (45.5%)	

The mean PSQI score was significantly higher in the asthmatic group compared to the control group (6.26 ± 2.01 vs. 3.41 ± 0.50; p-value: <0.0001). The percentage of participants with a PSQI score of ≥5 was significantly higher in the asthmatic group compared to the control group (54.5% vs. 17.0%; p-value: <0.0001) (Table 2).

**Table 2:** The comparison of PSQI score of both groups PSQI: Pittsburgh Sleep Quality Index

PSQI score	Case group (n=200)	Control group (n=200)	P-value
Mean score	6.26 ± 2.01	3.41 ± 0.50	<0.0001
Participants with ≥5 score	109 (54.5%)	34 (17.0%)	<0.0001

## Discussion

Our study indicates that the quality of sleep in the young asthmatic patients is impaired compared to the control group Mean PSQI score was higher in asthmatic patients compared to the control group. Our study’s mean score was found to be very close to Braido et al., which reported the mean score to be 5.68 ± 3.4 [11]. Moreover, a significantly higher number of asthmatic patients (54.5%) had a PSQI score of ≥5 compared to the control group (17%). Several similar studies in the past have shown results coinciding with our study, with a prevalence of high PSQI scores in asthmatic patients, such as 58.3% [11] and 60% [12].

Several studies have suggested that asthmatic patients are known to have troublesome nights, including not sleeping, waking up in the middle of the night, and early in the morning [7]. Recently, a study including a large sample size of approximately 5,000 patients from 10 European countries concluded that nasal and respiratory factors are correlated with a short duration of sleep (less than six hours) [13]. Disturbed sleep could potentially lead to drowsiness during the day. A Severe Asthma Research Program (SARP) cohort was conducted including 255 patients, 40% of patients with severe asthma presented with complaints of lethargy during the day, and 31% demonstrated a high Epworth Sleepiness Score [14]. This points toward the fact that the patients’ cognitive abilities and daytime working are affected; however, sufficient data on this aspect were not available as of now in adults. Fitzpatrick et al. analyzed 12 asthmatic patients focusing on their sleep patterns and including a broad spectrum of cognitive properties like the evaluation of short-term memory, focus, and patterns of attention [10]. Cukic et al. suggested that sleep disturbance in asthmatic patients can lead to daytime sleepiness and fatigue, and cognitive impairment [15]. In this small sample size, all patients with nocturnal asthma had disturbed intellectual performance. However, this aspect has been thoroughly investigated in children, and nocturnal asthma has been proven to disturb school performance and attendance.

Literature has made it clear that asthma negatively affects sleep quality. This, in turn, affects the quality functioning of the brain and daily day-to-day performance. Based on the results of our study, future studies are required to explore potential treatment options to improve the state of asthma. Improved asthma would eventually solve the problems related to sleep, leading to better cognitive and daily functioning. To the best of our knowledge, this is the first study in our regional setting to study the impact of asthma on sleep quality. However, since the study was conducted in a single institute and city, care should be taken while generalizing the result to a greater population.

## Conclusions

Our study indicates poor sleep among asthmatic patients. Poor sleep may interfere with their daily performance, which may further have a negative impact on QOL in asthmatic patients. To mitigate this problem, potential treatment options should be prescribed to help improve the severity of asthma. Treating the basic problem would eventually lead to the development of proper sleeping habits.

## References

[1] O'Byrne PM, Barnes PJ, Rodriguez-Roisin R, Runnerstrom E, Sandstrom T, Svensson K, Tattersfield A (2001). Low dose inhaled budesonide and formoterol in mild persistent asthma: the OPTIMA randomized trial. Am J Respir Crit Care Med.

[2] Masoli M, Fabian D, Holt S, Beasley R (2004). The global burden of asthma: executive summary of the GINA Dissemination Committee report. Allergy.

[3] Brehm JM, Celedón JC, Soto-Quiros ME (2009). Serum vitamin D levels and markers of severity of childhood asthma in Costa Rica. Am J Respir Crit Care Med.

[4] Sandhu MS, Casale TB (2010). The role of vitamin D in asthma. Ann Allergy Asthma Immunol.

[5] Hossny E, Caraballo L, Casale T, El-Gamal Y, Rosenwasser L (2017). Severe asthma and quality of life. World Allergy Organ J.

[6] Braido F, Baiardini I, Ghiglione V, Fassio O, Bordo A, Cauglia S, Canonica GW (2009). Sleep disturbances and asthma control: a real life study. Asian Pac J Allergy Immunol.

[7] Janson C, De Backer W, Gislason T (1996). Increased prevalence of sleep disturbances and daytime sleepiness in subjects with bronchial asthma: a population study of young adults in three European countries. Eur Respir J.

[8] Krouse HJ, Yarandi H, McIntosh J, Cowen C, Selim V (2008). Assessing sleep quality and daytime wakefulness in asthma using wrist actigraphy. J Asthma.

[9] Mastronarde JG, Wise RA, Shade DM, Olopade CO, Scharf SM (2008). Sleep quality in asthma: results of a large prospective clinical trial. J Asthma.

[10] Fitzpatrick MF, Engleman H, Whyte KF, Deary IJ, Shapiro CM, Douglas NJ (1991). Morbidity in nocturnal asthma: sleep quality and daytime cognitive performance. Thorax.

[11] Braido F, Baiardini I, Ferrando M (2021). The prevalence of sleep impairments and predictors of sleep quality among patients with asthma. J Asthma.

[12] Akinwalere OO, Adeniyi BO, Awopeju OF, Erhabor GE (2020). Impact of sleep quality on asthma control amongst asthmatics at Federal Medical Centre, Owo, Ondo State. West Afr J Med.

[13] Björnsdóttir E, Janson C, Lindberg E (2017). Respiratory symptoms are more common among short sleepers independent of obesity. BMJ Open Respir Res.

[14] Teodorescu M, Broytman O, Curran-Everett D (2015). Obstructive sleep apnea risk, asthma burden, and lower airway inflammation in adults in the Severe Asthma Research Program (SARP) II. J Allergy Clin Immunol Pract.

[15] Cukic V, Lovre V, Dragisic D (2011). Sleep disorders in patients with bronchial asthma. Mater Sociomed.

